# Supplementary material for: Sensory representations in the striatum provide a temporal reference for learning and executing motor habits
Source: Nat Commun. 2019 Sep 9;10:4074. doi: 10.1038/s41467-019-12075-y (PMC6733846; doi:10.1038/s41467-019-12075-y)
Supplement: Supplementary file 1 — Supplementary Information [file 41467_2019_12075_MOESM1_ESM.pdf]

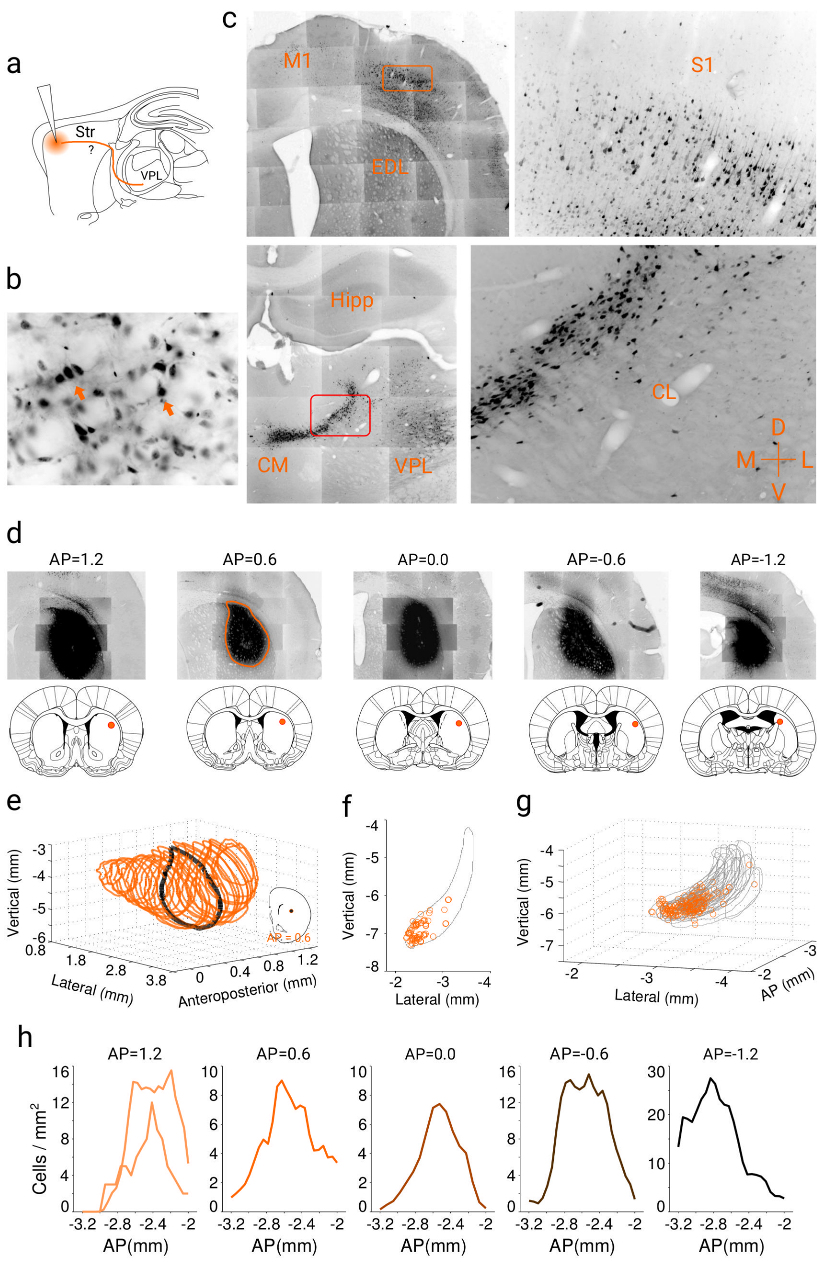

Supplementary Figure 1. Anatomical evidence of direct projections from the VPL to the DLS. (**a**) Schematic representation of the site of injection of the retrograde tracer Fluorogold (FG) in the dorsolateral striatum. (**b**) Amplified view (20x) of the VPL micrograph presented in Figure 1a; the arrows indicate examples of cells retrogradely labeled with FG. (**c**) Retrogradely labeled cells were also found in well-known sites projecting to the DLS, like S1 (Top) and the central medial/lateral nucleus of the thalamus (bottom). (**d**) Micrographs (top) and schematic representations (bottom) of the different sites in the DLS where FG was injected; each animal received only one injection. (**e**) 3D reconstruction of the total volume of diffusion of FG in the DLS in one animal injected at 0.6 mm anterior to bregma (indicated in orange in d). (**f**) The contours of the VPL were manually drawn (gray) and the number and positions of retrograde labelled cells (orange) were calculated for each slide (thickness = 50 mm). (**g**) An example of all slides and cells for one animal. (**h**) For each animal we plotted the distribution of cells in the anterior posterior axis of the VPL. The site of injection for each distribution is indicated at the top of each plot.

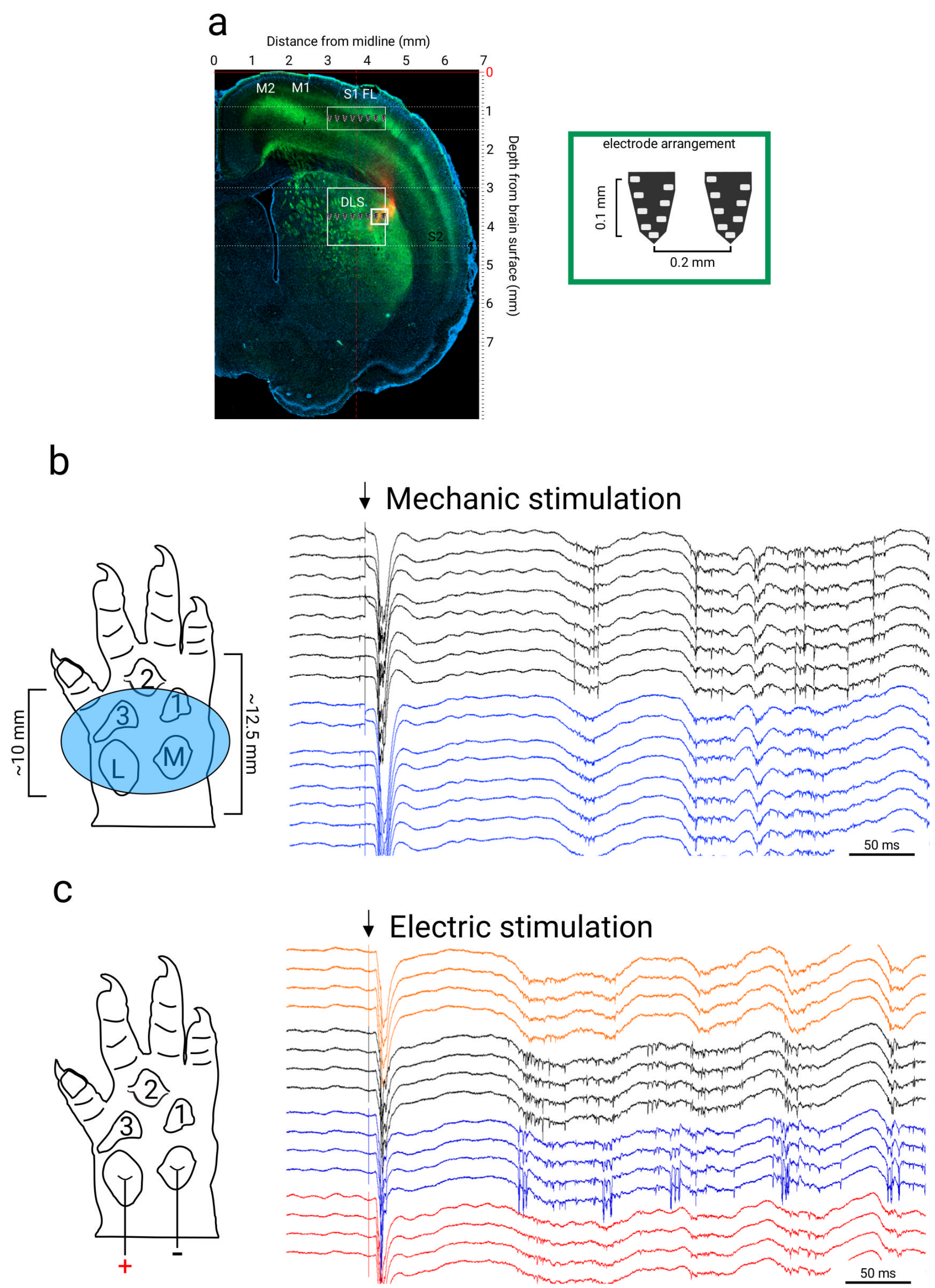

Supplementary Figure 2. Anesthetized recording conditions. (**a**) Representation of the recording sites in the forelimb region of the primary somatosensory cortex (S1 FL) and dorsolateral striatum (DLS). Recordings were performed with 64-channel silicon probes divided in 8 shanks (8 electrodes on each shank). The dimensions of each shank tip and the position of the electrodes are drawn on the right. The entire array was centered in the middle of the forelimb region of S1 and covered a 1.4 mm area on the horizontal plane. Schematic representation of the area stimulated on the palm of a forepaw (**b,** left) with a cotton tip (blue) or electrical stimulation with insertion electrodes in the middle and lateral pads (**c**, left). Representative traces (16 channels) of a typical somatosensory evoked response in S1after mechanical (b, right) or electrical stimulation (c, right).

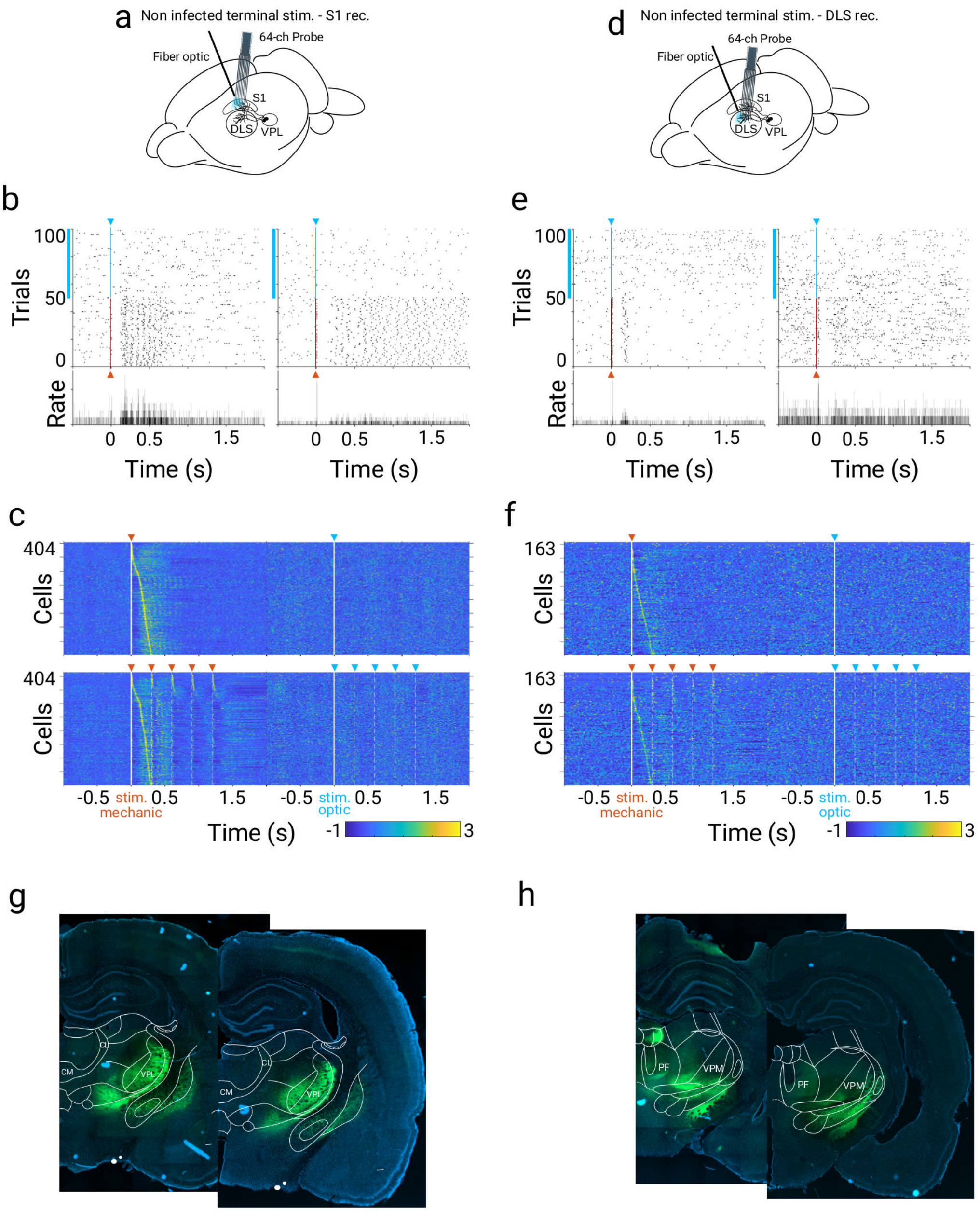

Supplementary Figure 3. Light stimulation in non-infected animals did not evoke cortical or striatal responses. Non-infected animals were recorded and stimulated in S1 (**a**) and DLS (**d**). Spike rasters (top) and average peri-event histograms (bottom) of two illustrative units aligned to the onset of mechanical (trials 1-50; orange code) and optical (trials 51-100; blue code) stimulation of the VPL (**b**) and S1 (**e**). Averaged firing rates for cells recorded in S1 (**c**) and DLS (**f**) after mechanic (orange arrows) or optical stimulations (blue arrows). Activity is expressed as Z-score (color coded bar) and neurons are sorted according to the time they reached the highest activity after stimulus onset. Stimulation was given as single stimulus (upper panels) or trains of stimulus (lower panels). Representative sections where the CM/CL complex (g) and the parafasicular nucleus are present (h). The CL/CM rarely showed fluorescent terminals. On the contrary, the parafasicular nucleus regularly contained fluorescent staining consistent with terminals from the VPL.

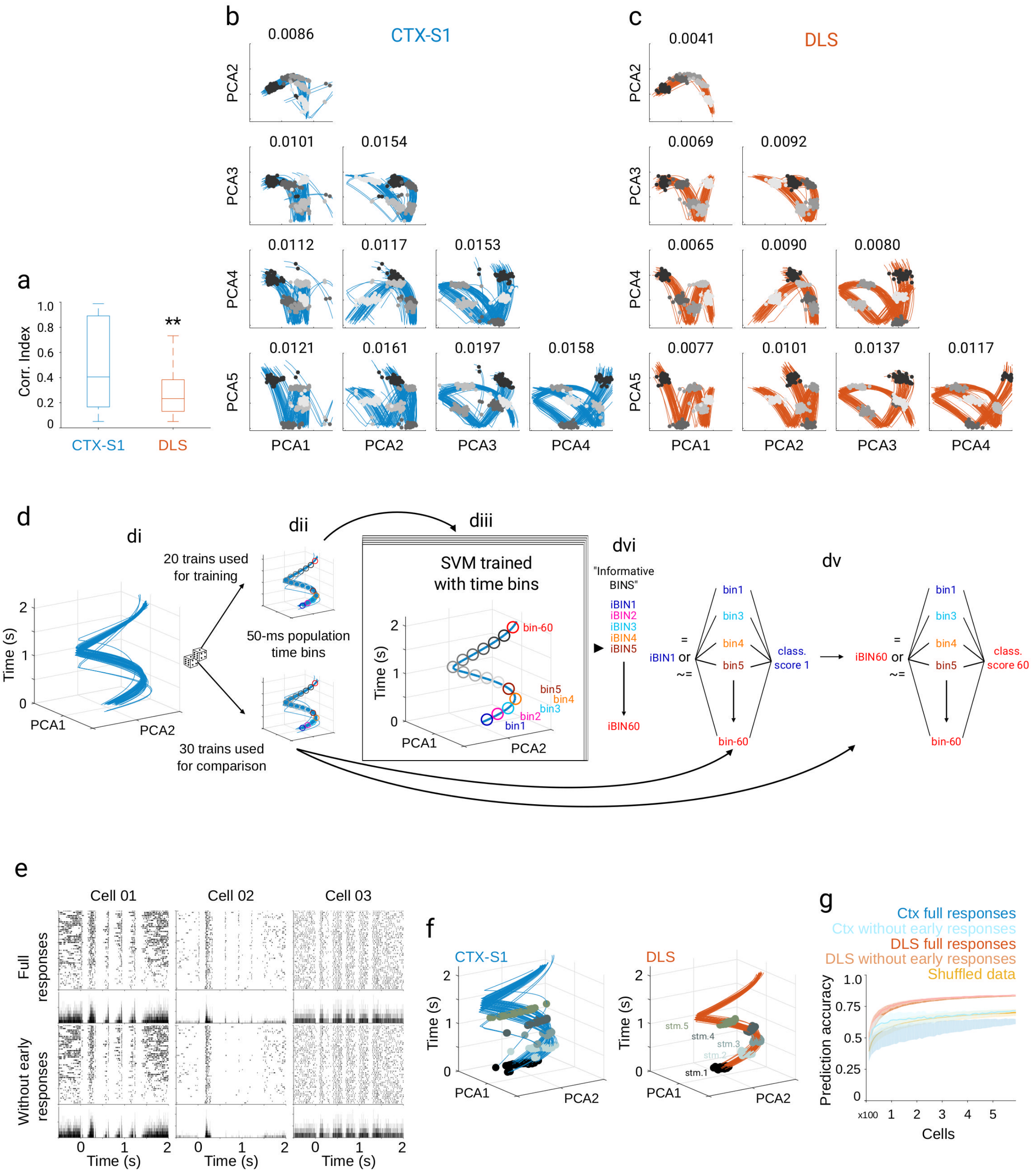

Supplementary Figure 4. Population representations of somatosensory information in S1 and DLS. (**a**) Correlation values between population trajectories evoked by every stimulus of the train in S1 (blue) and DLS (orange). Box plots represent median and 25th and 75th percentiles. Low-dimensional projection of the population activity for all possible combinations of PCAs 1 to 5 in S1 (**b**) and DLS (**c**) evoked by 50 stimulation trains. (**d**) Schematic representation of SVM procedure to decode elapsed time. (**di**) PCA population trajectories were randomly assigned for training (20 population trajectories) or comparison (30 population

trajectories). (dii) Each population trajectory was binarized (50-ms time bins). (**diii**) SVM binary classifiers are trained to identify the population activity pattern for each time bin. (**div**) As a result SVM produced 60 “informative bins”, one for each time bin. (**dv**) Finally, the activity patterns from the remaining 30 comparison population trajectories (dii) are compared with the informative bins (dvi). (**e**) Spike rasters (top) and average peri-event histograms (bottom) for 3 representative units before (upper row) and after (lower row) artificially removing spikes from the 50-ms immediately after each stimulus of every train. (**f**) Low-dimensional projection of the population activity in S1 (left) and DLS (right) from the “modified trains” (e). (**g**) Same as in Figure 3d but using the activity from “modified trains”.

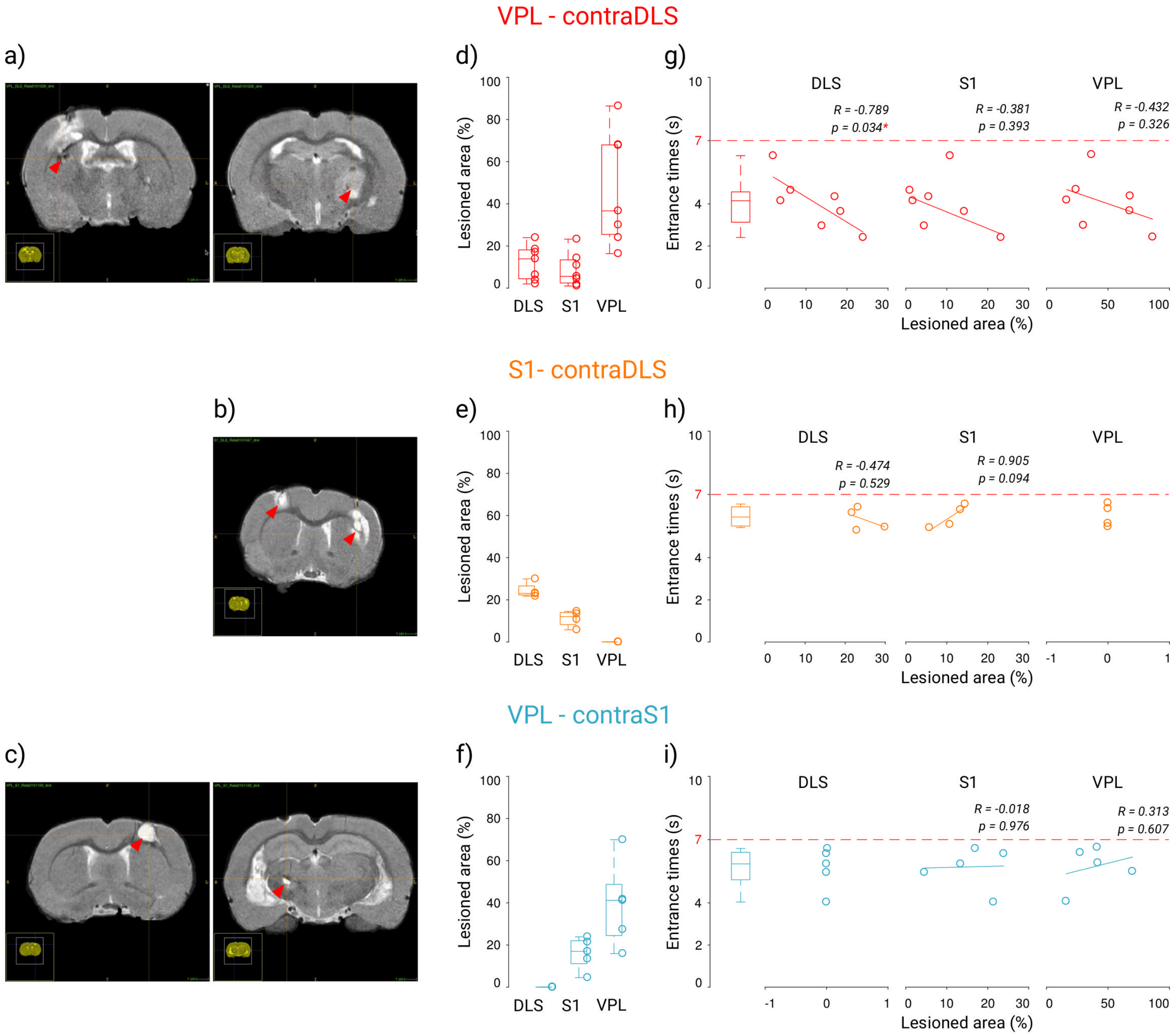

Supplementary Figure 5. Estimation of damaged tissue. Magnetic resonance coronal slices for representative animals in the DLS-VPL (**a**), S1-DLS (**b**) and S1-VPL (**c**) groups obtained by structural magnetic resonance imaging. Lesion areas indicated by arrowheads. (**d**, **e**, **f**) Percentage of damaged tissue for each structure in each group; median 25th and 75th percentiles and individual cases (circles) are presented. (**g**, **h**, **i**) Correlation plots between performance, represented as median 25th and 75th percentiles of the entrance times during the first 20 sessions of training, and the percentage of damaged tissue in each structure. Multiple correlation analysis was also performed confirming that the combination of lesions VPL+DLS (R = 0.89; *p* = *0.0107*) is the configuration that better explains the data. VPL+DLS+S1 (R = 0.94; *p*= *0.0224*); DLS+S1 (R = 0.66; *p* = *0.114*); VPL + S1 (R = 0.19; *p=0.6518*).

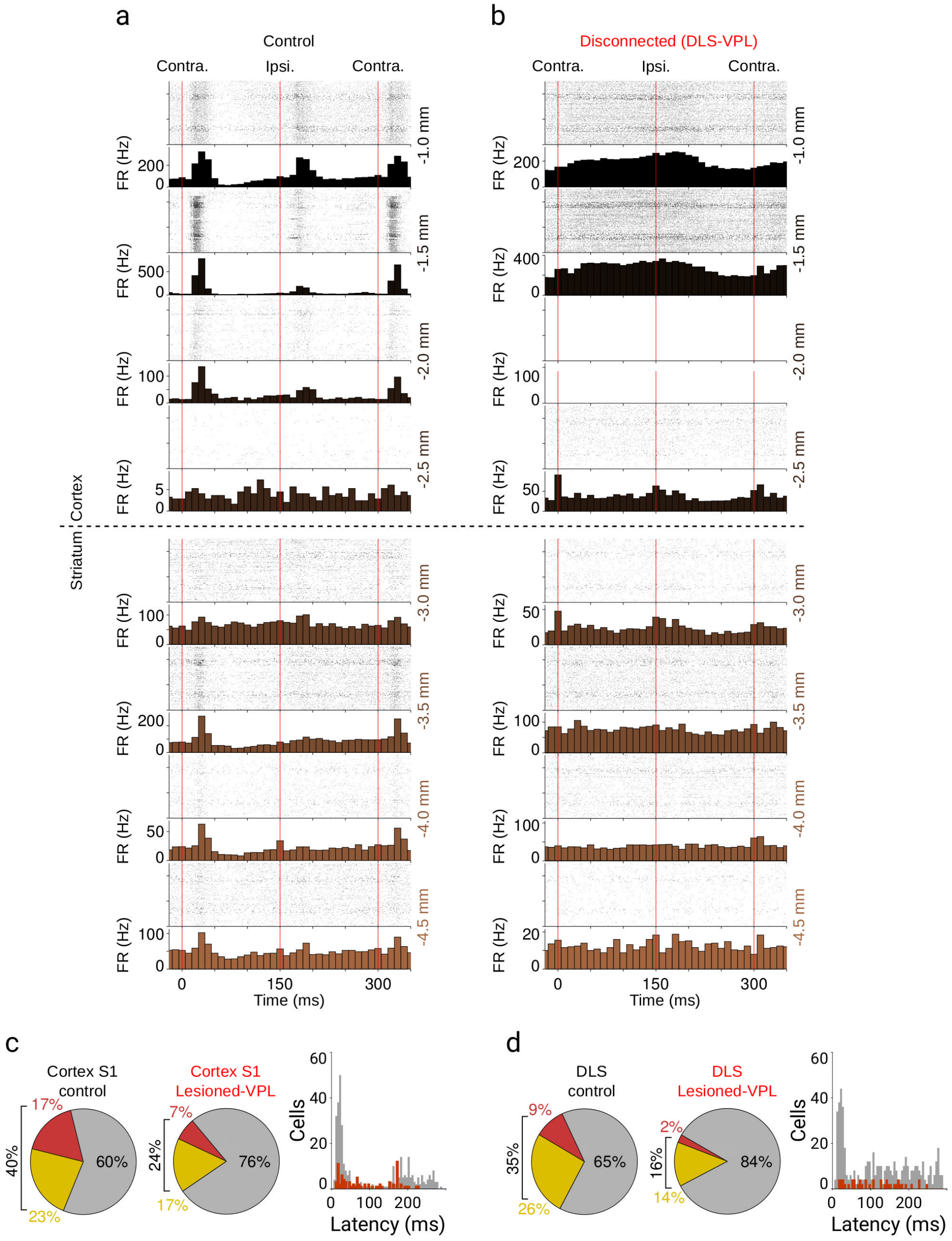

Supplementary Figure 6. Lesions of the VPL remove sensory representations of the forelimb in S1 and DLS. Activity of fifteen illustrative cells from control (a) and VPL lesioned (b) animals were aligned to the stimulation onset of the first stimulus of the train. Stimulation in both forelimbs was applied in these recordings. Five stimuli for each forelimb were delivered at 3.3 Hz and 150 ms out of phase between the contralateral and ipsilateral stimulations. Spike rasters (top) and average peri-event histograms (bottom) are depicted for cells recorded at different depths (indicated on the right of each plot) from the surface of the brain. Cortical and striatal cells above and below dotted line respectively. (**c**, **d**) Pie plots representing the percentage of cells that significantly changed their firing rates during the first 30ms (red) or 300 ms (yellow) after the first stimulus of the train in the cortex (c) and striatum. Histograms of the response latencies for all cells recorded in S1 (black histogram) and DLS (orange histogram) of VPL lesioned animals.

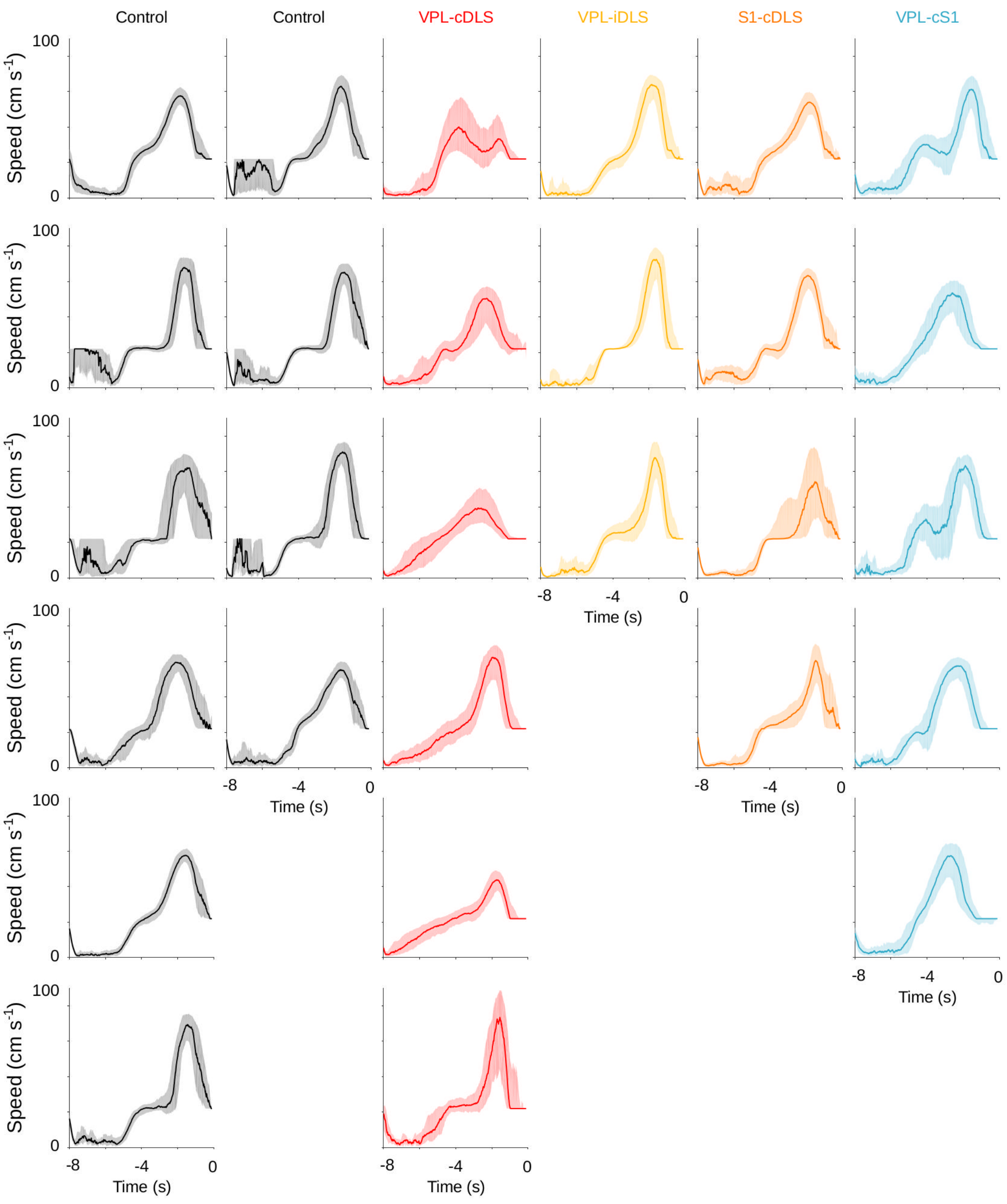

Supplementary Figure 7. Speed trajectories. Speed trajectories (median + 75th & 25th percentiles) for all animals (color code as in Figure 4).

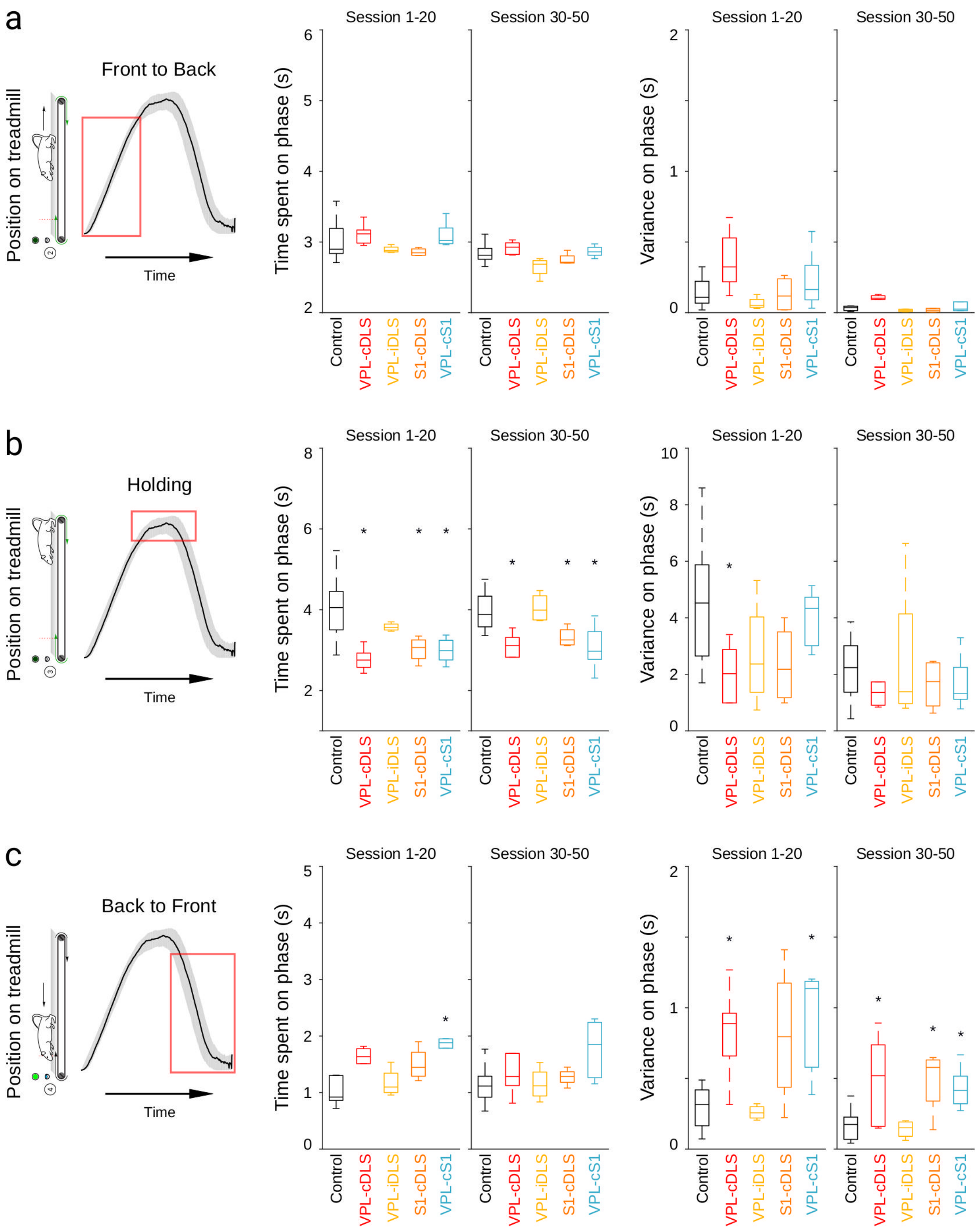

Supplementary Figure 8. Motor sequence analysis. Time (middle panels) and Variance (right panels) for the Front-to-Back (**a**), Holding time (**b**) and Back-to-Front (**c**) phases of the sequences for all groups trained under different lesion arrangements (color code as in Figure 4). Data are presented as median + 75th and 25th percentiles for the grouped early (sessions 1 - 20) or late (sessions 30 - 50) sessions of training. Significant differences were obtained with Kruskal-Wallis and Bonferroni post hoc test * $p < 0.05$. a) early times $X^2 = 8.66$, p=0.073; late times $X^2 = 10.01$ p=0.056; early variances $X^2 = 9.39$, p=0.052; late variances $X^2 = 9.28$ p=0.054. b) early times $X^2 = 16.33$, p=0.002; late times $X^2 = 16.75$ p=0.002; early variances $X^2 = 9.6$, p=0.047; late variances $X^2 = 1.61$ p=0.806. c) early times $X^2 = 10.45$, p=0.033; late times $X^2 = 6.75$ p=0.149; early variances $X^2 = 12.72$, p=0.012; late variances $X^2 = 13.55$ p=0.008.

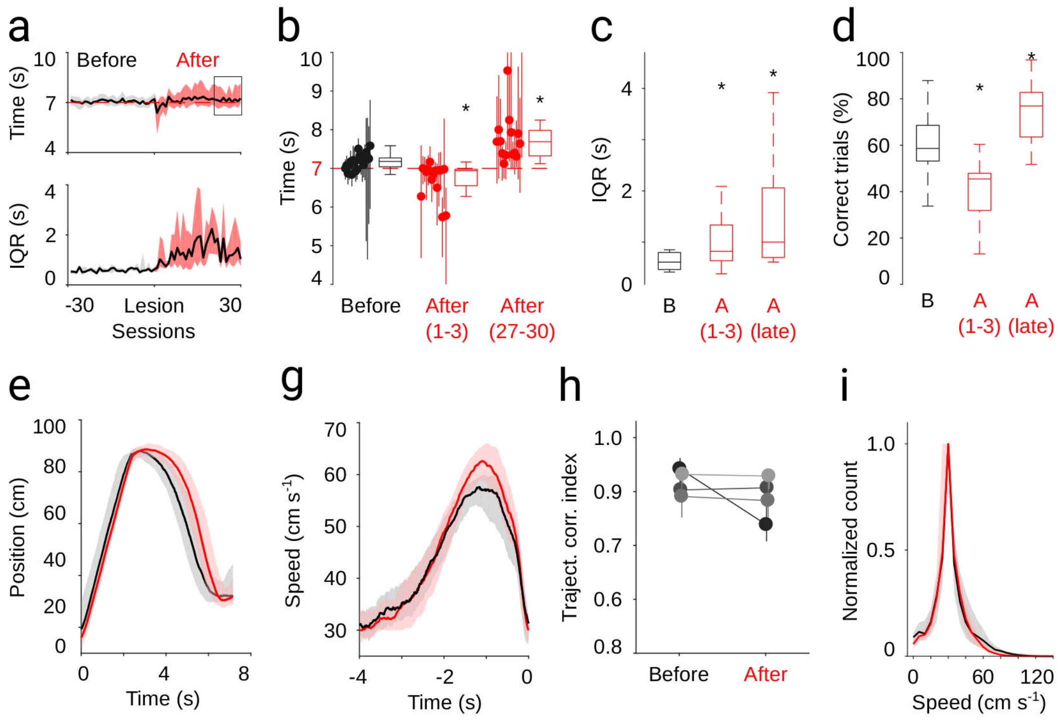

Supplementary Figure 9. Functional disconnection of the VPL-DLS pathway in expert animals. (**a**) Goal area entrance times (top; median 25th & 75th percentiles) and interquartile range (top; median 25th & 75th percentiles) for the last and first 30 sessions before (black) and after (red) the functional disconnections of the VPL-DLS pathway. (**b**) Goal area entrance time (median 25th & 75th percentiles) sessions before and early after (first 3 sessions) and late after (last 10 sessions, indicated with square) functional disconnections. Dots and lines are individual sessions for each of the 5 animals. (**c**, **d**) Interquartile range (**c**) and percentage of correct trials (**d**) for the same sessions as in b. (**e** – **g**) Representative average position (**e**) and speed (**g**) trajectories of one animal in 10 sessions before (black) and 10 after (red, square in a) disconnection experiments. (**h**) Trajectory correlation index from the same sessions as "e" for all animals. (**i**) Normalized distribution of running speeds (median + 75th & 25th percentiles) expressed in the punishment periods of incorrect trials for sessions before (black) and after (red) functional disconnections. * Kruskal-Wallis and Tukey's HSD test; $p < 0.01$.

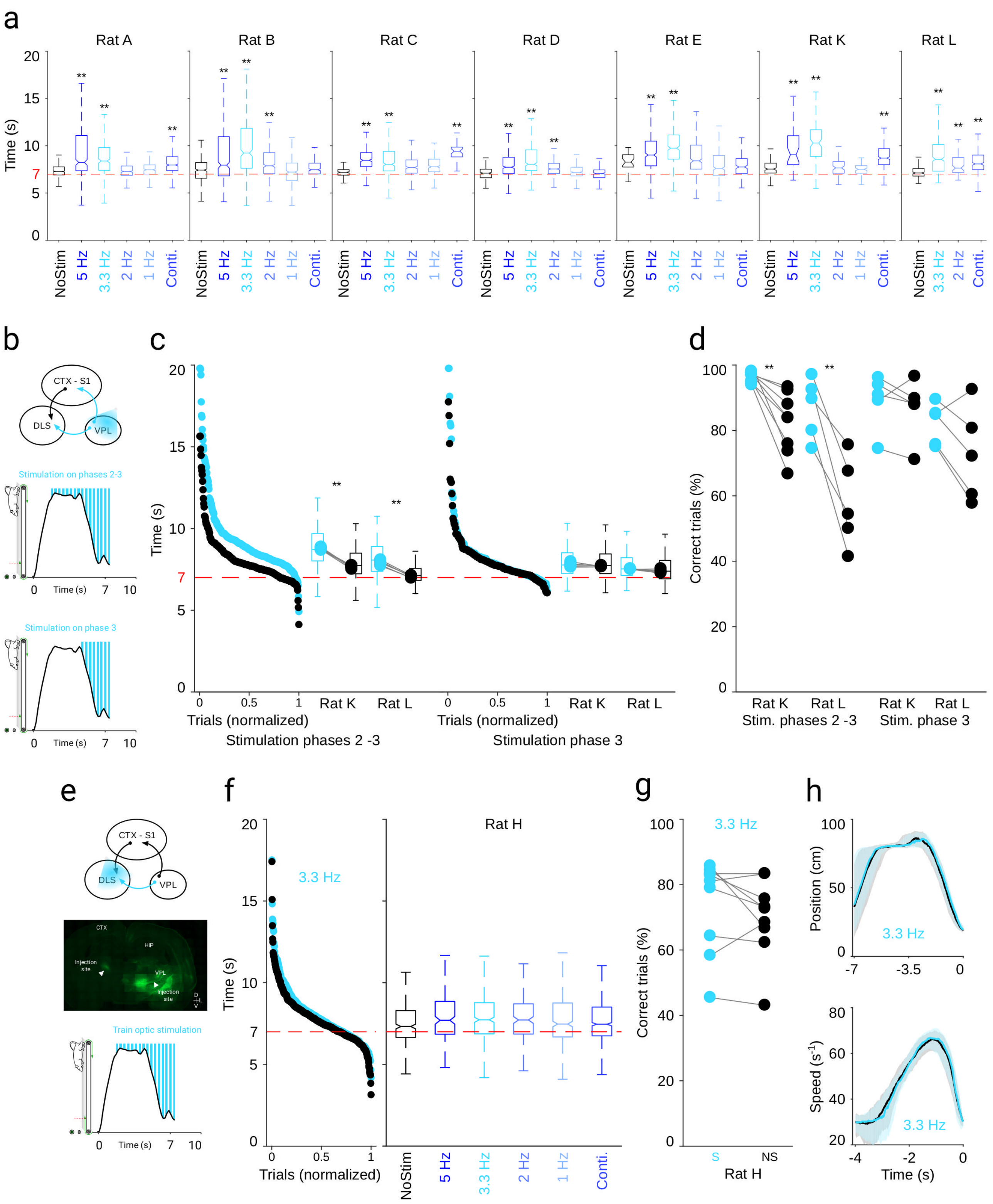

Supplementary Figure 10. Optogenetic manipulations. (**a**) Entrance times for all stimulation protocols of the animals stimulated with Channelrhodopsin 2. (**a, e**) Schematic representation of the site of infection and stimulation (b, e, top). Light stimulation was delivered in trains starting at the beginning of the holding phase (b, e middle; "phases 2-3") or only during the last acceleration phase (b, bottom; "phase 3") of the sequences in 50% of the randomly selected trials. Entrance times (c) and percentage of correct responses (d) for all stimulated (blue) vs

non-stimulated (black) trials for Rats Kand L when stimulation was delivered in phases 2-3 (left) or only in phase 3 (right). Histological confirmation of viral expression for Rat H (**e**, middle). Entrance times (**f**), percentage of correct responses (**g**), average position (**h,** top) and speed (**h**, bottom) trajectories during stimulated (blue) vs non-stimulated (black) trials for Rat H. Box plots indicate the median (25th & 75th percentiles); pairs of dots united by lines indicate medians of each session per animal. ** in panel a, Kruskal-Wallis and Tukey's HSD test; $p < 0.001$; ** in panels c and d, Wilcoxon rank-sum test.

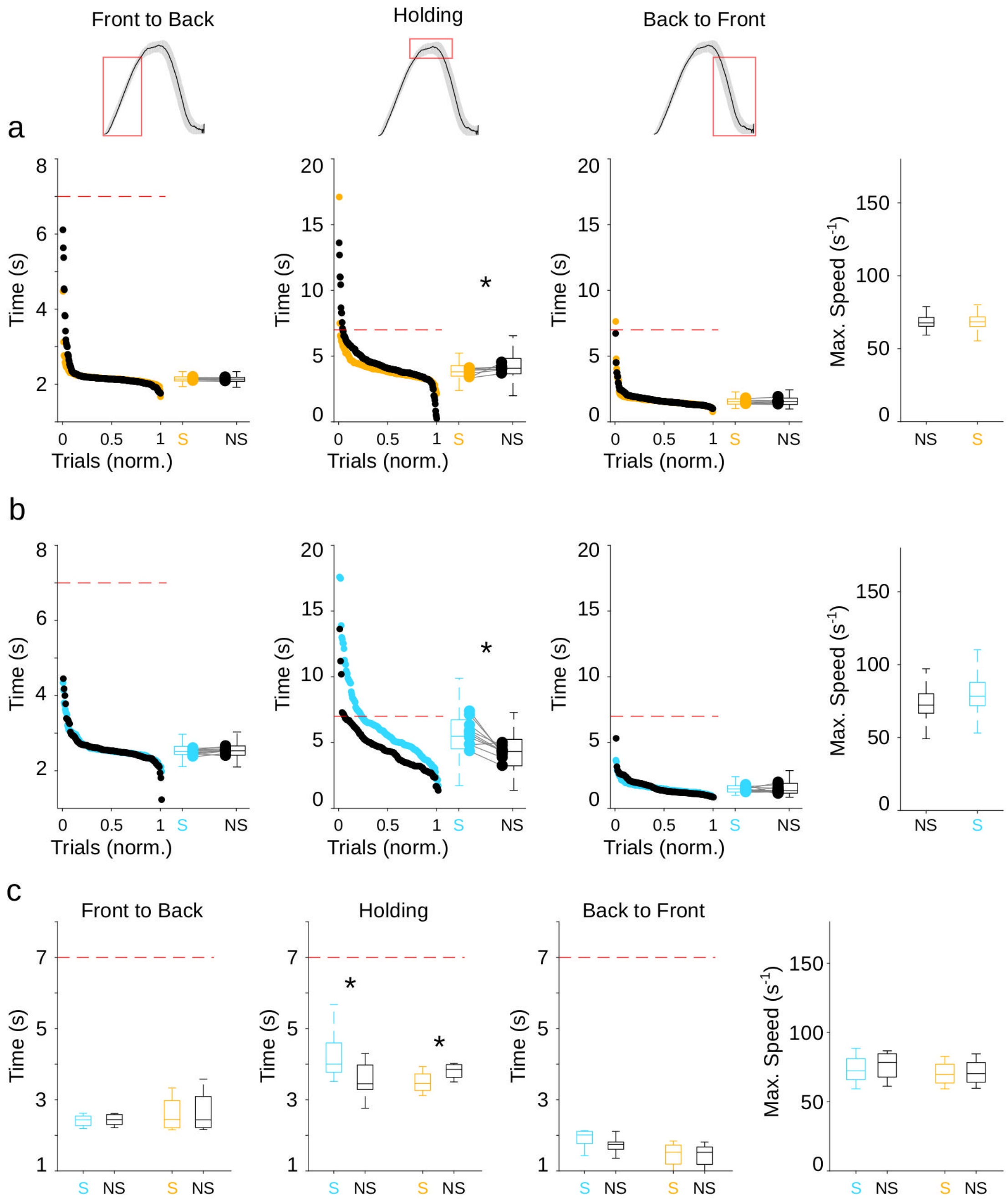

Supplementary Figure 11. Motor sequence analysis in optogenetic experiments. From left to right, times spent in the Front-to-Back, Holding and Back-to-Front phases of the sequences and maximum speeds expressed during stimulated (yellow/blue) and non-stimulated (black) trials for two representative animals infected with Archaerhodopsin (**a**) and Channelrhodopsin 2 (**b**) in the VPL. Schematic representations of the different phases of the sequence are depicted in the upper part of the figure. (**c**) Median + 75th and 25th percentiles for the grouped data of all animals (animals stimulated in the VPL or its terminals were grouped together). Significant differences were obtained with Wilcoxon rank-sum test; * $p < 0.05$.

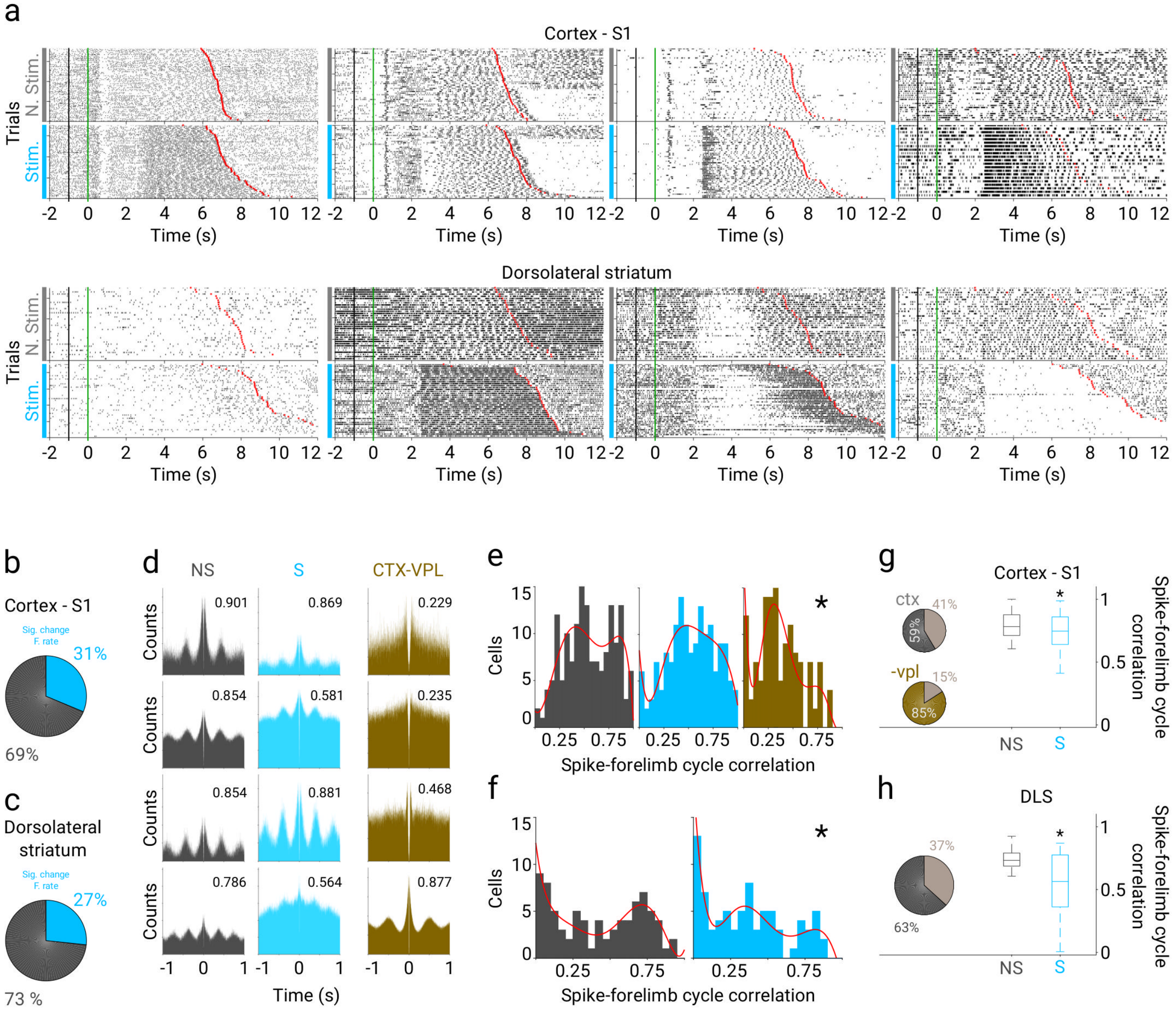

Supplementary Figure 12. Freely moving corroboration of optogenetic manipulations. (a) Activity of eight illustrative units aligned to treadmill onset (green line) recorded in S1 (upper rows) or DLS (lower rows). Stimulated (blue) and non-stimulated (gray) trials are represented in the upper and lower part of the rasters and signaled by a color bar on the left of each panel. Trials were sorted according to the entrance times (red dots). (**b**, **c**) Percentage of cells that significantly changed their firing rate between stimulated and non-stimulated trials in S1 (**b**) and DLS (**c**). (**d**) First two columns, autocorrelograms of four representative neurons (rows) recorded during the execution of the motor sequence in non-stimulated (gray) and stimulated (blue) trials. In the last column, four representative autocorrelograms from representative cells recorded during the execution of the task in one animal subjected to a pharmacological lesion of the VPL (ipsilateral to the recording site) and the contralateral DLS. Correlation values with the forelimb cycle are displayed on each autocorrelogram. Spike-Forelimb correlation distributions for cells recorded in S1 (e) and DLS (f). Red lines correspond to a 5th order polynomial fit. Color code as in “d”. (**g, h**) Left, percentage of cells with correlation values higher than 0.6 in S1 (g) and DLS (h). Right, box plots showing median 25th & 75th percentiles of the same cells during non-stimulated (gray) vs stimulated (blue) trials. Significant differences were obtained with Wilcoxon rank-sum test (e) and Wilcoxon signed-rank-sum test (f, g, h); * $p < 0.05$.
