# Supplementary material for: Sensory representations in the striatum provide a temporal reference for learning and executing motor habits
Source: Nat Commun. 2019 Sep 9;10:4074. doi: 10.1038/s41467-019-12075-y (PMC6733846; doi:10.1038/s41467-019-12075-y)
Supplement: Supplementary file 3 — Reporting Summary [file 41467_2019_12075_MOESM3_ESM.pdf]

nature research

Corresponding author(s): Pavel E Rueda-Orozco

Reporting Summary

Nature Research wishes to improve the reproducibility of the work that we publish. This form provides structure for consistency and transparency in reporting. For further information on Nature Research policies, see Authors & Referees and the Editorial Policy Checklist.

Statistical parameters

When statistical analyses are reported, confirm that the following items are present in the relevant location (e.g. figure legend, table legend, main text, or Methods section).

n/a	Confirmed	
☐	☒	The exact sample size (*n*) for each experimental group/condition, given as a discrete number and unit of measurement
☐	☒	An indication of whether measurements were taken from distinct samples or whether the same sample was measured repeatedly
☐	☒	The statistical test(s) used AND whether they are one- or two-sided<br>*Only common tests should be described solely by name; describe more complex techniques in the Methods section.*
☒	☐	A description of all covariates tested
☐	☒	A description of any assumptions or corrections, such as tests of normality and adjustment for multiple comparisons
☐	☒	A full description of the statistics including central tendency (e.g. means) or other basic estimates (e.g. regression coefficient) AND variation (e.g. standard deviation) or associated estimates of uncertainty (e.g. confidence intervals)
☐	☒	For null hypothesis testing, the test statistic (e.g. *F*, *t*, *r*) with confidence intervals, effect sizes, degrees of freedom and *P* value noted<br>*Give P values as exact values whenever suitable.*
☒	☐	For Bayesian analysis, information on the choice of priors and Markov chain Monte Carlo settings
☒	☐	For hierarchical and complex designs, identification of the appropriate level for tests and full reporting of outcomes
☒	☐	Estimates of effect sizes (e.g. Cohen's *d*, Pearson's *r*), indicating how they were calculated
☐	☒	Clearly defined error bars<br>*State explicitly what error bars represent (e.g. SD, SE, CI)*

Our web collection on statistics for biologists may be useful.

Software and code

Policy information about availability of computer code

Data collection	Behavior was monitored, acquired and analyzed using custom-made routines in LabVIEW environment from National Instruments. Electrophysiological data was acquired with custom-made routines in LabView environment from National Instruments or the “Open-Source RHD2000 Interface Software” from Intan Tehcnologies (http://intantech.com).
Data analysis	Electrophysiological data was processed with the open-source software Klusters, Neuroscope and NDManager developed by Lynn Hazan and Michael Zugaro (http://neurosuite.sourceforge.net) and KlustaKwik developed by Ken Harris (http://klustakwik.sourceforge.net). Further analysis for electrophysiological and behavioral data was performed with Matlab (MathWorks) statistical toolbox.

For manuscripts utilizing custom algorithms or software that are central to the research but not yet described in published literature, software must be made available to editors/reviewers upon request. We strongly encourage code deposition in a community repository (e.g. GitHub). See the Nature Research guidelines for submitting code & software for further information.

Data

Policy information about availability of data

All manuscripts must include a data availability statement. This statement should provide the following information, where applicable:

- Accession codes, unique identifiers, or web links for publicly available datasets
- A list of figures that have associated raw data
- A description of any restrictions on data availability

The datasets generated during the current study are available from the corresponding author on reasonable request

Field-specific reporting

Please select the best fit for your research. If you are not sure, read the appropriate sections before making your selection.

- [x] Life sciences
- [ ] Behavioural & social sciences
- [ ] Ecological, evolutionary & environmental sciences

For a reference copy of the document with all sections, see nature.com/authors/policies/ReportingSummary-flat.pdf

Life sciences study design

All studies must disclose on these points even when the disclosure is negative.

Sample size	No statistical method was used to determine the sample size. Sample size for each experiment was determined based on previous reports. For electrophysiological experiments in anesthetized animals we report the number of cells (always in the range of hundreds). By using multielectrode arrays and offline spike sorting we collected similar size samples than previous reports using similar techniques (e.g. Mochol et al, 2012; Bermudez-Contreras et al, 2013) and at least duplicate sample sizes of previous reports using other techniques (e.g. Shin & Chapin, 1990; Francis & Chapin, 2008; Smith et al, 2012). For behavioral experiments, the design of the task allowed us to generate thousands of trials for each animal, increasing the statistical power. For the behavioral manipulations we report sample sizes similar (but higher) than previous reports using the same task (Rueda-Orozco et al, 2015). Finally, the long training periods for each animal (> 10, 000 trials in > 4 months) also justified the relative low number of animals.
Data exclusions	No data were excluded from the analysis
Replication	Electrophysiological and behavioral observations were robust and clearly present in all animals as stated throughout the results section. Nevertheless, in a single animal the behavioral outcome elicited by optogenetic manipulations of the VPL-DLS terminals was virtually absent (Rat H, on Supplementary figure 10 e-h and last paragraph in results section). However, this result was completely explained by a mistake in the coordinates of the infection site in the thalamus.
Randomization	For behavioral experiments where animals were lesioned before training (figure 4), animals were allocated in control and experimental groups by simple randomization. For the rest of behavioral observations, manipulations were performed after long periods of overtraining, and we report both conditions before, during and after manipulations for all animals. In this case no randomization was necessary since every animal served as their own control.
Blinding	Data collection could not be performed blindly. However, for behavioral experiments, the experimenter was not present in the experimental room during data collection. For electrophysiological experiments analysis of the spiking activity and behavior was performed offline and Matlab programs were run in batch on all the data independently of experimental conditions.

Reporting for specific materials, systems and methods

Materials & experimental systems

n/a	Involved in the study	
x		Unique biological materials
x		Antibodies
x		Eukaryotic cell lines
x		Palaeontology
	x	Animals and other organisms
x		Human research participants

Methods

n/a	Involved in the study	
x		ChIP-seq
x		Flow cytometry
	x	MRI-based neuroimaging

Animals and other organisms

Policy information about studies involving animals; ARRIVE guidelines recommended for reporting animal research

Laboratory animals	We used Long-Evans rats (n = 61; 250g – 700g). Animlas were housed in pairs at a stable temperature (23 oC) and humidity (66%) under a constant 12:12-h light-dark cycle (lights on at 8 am) and with free access to food and water.
Wild animals	The study did not involve wild animals
Field-collected samples	The study did not collected samples from the field

Magnetic resonance imaging

Experimental design

Design type	There was no experimental design, only structural images were acquired.
Design specifications	n/a
Behavioral performance measures	n/a

Acquisition

Imaging type(s)	Structural
Field strength	Bruker Pharmascan 70/16US, 7 Tesla MR scan
Sequence & imaging parameters	The anatomical scans were acquired using a spin-echo rapid acquisition with refocused echo (Turbo-RARE) sequences with the following parameters: repetition time = 1800 ms; echo time = 38 ms; RARE factor = 16; number of averages = 2; field of view = 18 X 20 mm2; matrix dimension = 144 X 160; slice thickness = 0.5 mm, resulting in voxel size of 0.08 x 0.08 x 0.5 mm3
Area of acquisition	With the help of the Paxinos & Watson atlas, for each animal we manually centered the scan area to include the totality of our target structures, i.e. the striatum, the primary somatosensory cortex (forelimb region) and the VPL. We scanned an area of approximately 9.5 mm from 2.5 mm anterior to -7 mm posterior with respect to bregma.

Diffusion MRI ☐ Used ☒ Not used

Preprocessing

Preprocessing software	MRI T2 images were pre-processed with a free code developed by Coupé et al., 2008 and Tuistison et al., 2010, which applies non-local means denoising using minc-toolkit and the N4 bias field correction from ANTs. After pre-processing, manual segmentation of unilateral lesions was performed with ITK snap to create masks of each region of interest (DLS, S1, VPL). The lesioned area was identified by hyperintensity voxel change compared with the non-lesioned counterpart. Finally, lesion volume quantifications were performed with Rstudio
Normalization	n/a
Normalization template	n/a
Noise and artifact removal	n/a
Volume censoring	n/a

Statistical modeling & inference

Model type and settings	n/a
Effect(s) tested	n/a

Specify type of analysis: ☒ Whole brain ☐ ROI-based ☐ Both

Statistic type for inference (See Eklund et al. 2016)	*Specify voxel-wise or cluster-wise and report all relevant parameters for cluster-wise methods.*
Correction	*Describe the type of correction and how it is obtained for multiple comparisons (e.g. FWE, FDR, permutation or Monte Carlo).*

Models & analysis

n/a	Involved in the study
☒	☐ Functional and/or effective connectivity
☒	☐ Graph analysis
☒	☐ Multivariate modeling or predictive analysis
